# Integrated High-Temporal-Resolution and High-Density Subretinal Prosthesis Using a Correlated Double-Sampling Technique

**DOI:** 10.3390/s23146501

**Published:** 2023-07-18

**Authors:** Hosung Kang, Jungyeon Kim, Jungsuk Kim

**Affiliations:** 1 Department of Medical Science, Korea University, Seoul 02841, Republic of Korea; 2017010569@korea.ac.kr; 2 Department of Health Sciences & Technology, Gachon Advanced Institute for Health Sciences & Technology, Gachon University, Incheon 21936, Republic of Korea; kjyeon@cellico.com; 3 Cellico Research and Development Laboratory, Sungnam-si 13449, Republic of Korea; 4 Department of Biomedical Engineering, Gachon University, Incheon 21936, Republic of Korea

**Keywords:** subretinal prosthesis, image sensor, high-density pixels, neural stimulator, implantable device, temporal resolution, correlated double sampling, current amplifier

## Abstract

This paper presents a 1600-pixel integrated neural stimulator with a correlated double-sampling readout (DSR) circuit for a subretinal prosthesis. The retinal stimulation chip inserted beneath the photoreceptor layer comprises an array of an active pixel sensor (APS) and biphasic pulse shaper. The DSR circuit achieves a high signal-to-noise ratio (SNR) of the APS with a short integration time to simultaneously improve the temporal and spatial resolutions of restored vision. This DSR circuit is adopted along with a 5 × 5-pixel tile, which reduces pixel size and improves the SNR by increasing the area occupied by storage capacitors. Moreover, a low-mismatch reference generator enables a low standard deviation between individual pulse shapers. The 1600-pixel retinal chip, fabricated using the 0.18 μm 1P6M CMOS process, occupies a total area of 4.3 mm × 3.3 mm and dissipates an average power of 3.4 mW; this was demonstrated by determining the stimulus current patterns corresponding to the illuminations of an LCD projector. Experimental results show that the proposed high-density stimulation array chip can achieve a high temporal resolution owing to its short integration time.

## 1. Introduction

**Figure 1:**
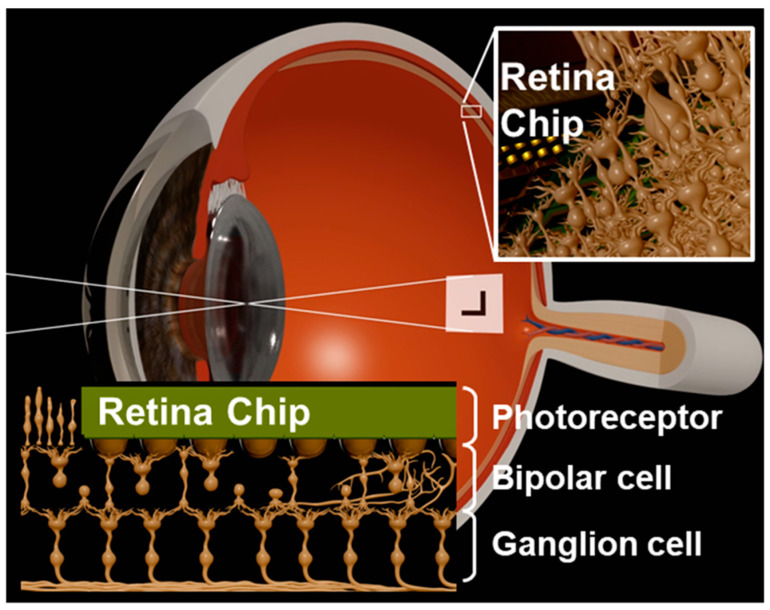
Concept of a subretinal prosthesis implanted beneath a bipolar cell.

Retina degeneration caused by rhodopsin mutation disrupts the photo-transduction process of converting incident light into electrophysiological spikes in the photoreceptor layer [1,2,3]. Over the last decade, retinal prostheses have been proposed to elicit the electrical conduction of the retinal signaling pathway (Figure 1). A conventional retina prosthesis inserted into the epi- or sub-retinal space of an eye functions as both an image sensor and a neural stimulator [4,5,6,7].

Among various retina prostheses, the sub-retinal prosthesis, which can insert an image sensor chip beneath the bipolar layer of the retina, can achieve a high-density neural stimulator array of 1600 pixels in a limited chip area of 5 mm × 5 mm [8,9]. This is achieved using an active pixel sensor (APS) to directly convert incident light into electrical stimulation without requiring any intermediate circuits, such as a high-resolution current steering digital-to-analog converter (DAC) and its local digital controller [10,11]. However, common subretinal chip schemes have challengeable design problems. First, during the stimulation of multiple pixels in a confined retinal area, interference occurs between narrow neighboring pixels, resulting in a blurry image. Second, to avoid the blur problem, each pixel must be stimulated individually. This causes a low temporal resolution of stimulation patterns. 

Motivated by this, this paper proposes a high-temporal resolution and 1600-pixel subretinal stimulator, which was designed and fabricated using a DB-HiTeK 0.18 *µ*m standard process. The proposed retina chip was implemented using a correlated double-sampling-based V-I converter circuit that resolves both problems of the stimulus temporal resolution and image blur. The temporal resolution, also known as the stimulation period, is determined by the integration time of the APS and the stimulus duration of the current pulse shaper (PS). Here, to achieve a high temporal resolution while maintaining the efficacy of electrical stimulation, it is necessary to reduce the input-referred noise of the readout circuit to achieve a high signal-to-noise ratio (SNR) from a signal of the APS [12]. Therefore, we adopted the correlated double sampling (CDS) technique, which mitigates electronic noise to acquire a light-induced voltage shift within a short integration time and enables a high temporal resolution [13,14].

The remainder of this paper is organized as follows. Section 2 presents an implementation of the high-density retina chip: (1) a sensor pixel of the APS and a PS of the current stimulator; (2) a CDS-based readout circuit to obtain a light intensity through the sensor pixel; (3) its peripheral circuits. Section 3 displays the measurement results of the retina chip. Section 4 concludes the paper, with a discussion and future work.

## 2. Circuit Implementation

**Figure 2:**
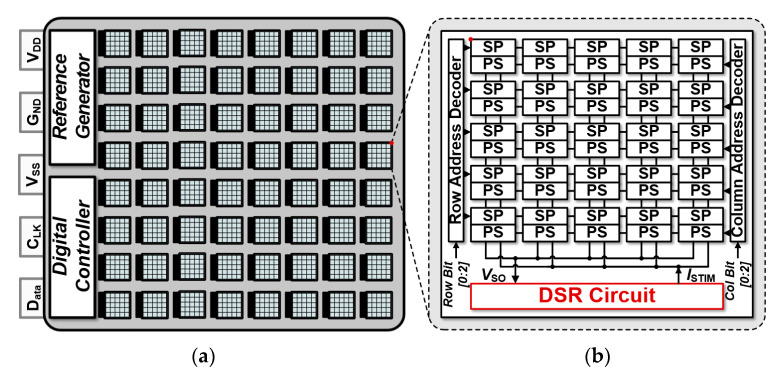
(**a**) Proposed 1600-pixel retina chip; (**b**) 5 × 5 pixel tile coupled with the DSR circuit.

Figure 2a shows block diagrams of the proposed 1600-pixel retinal chip architecture. A reference generator supplies a biasing current to readout circuits. A digital controller generates a global digital signal such as an integration time, a stimulation duration, and address bits. A single pixel in Figure 2b is composed of a sensor pixel (SP), detecting an incident light intensity during the integration time, and a pulse shaper (PS), generating a biphasic current pulse depending on a stimulation current I_STIM_. A pixel tile contains 25 pixels of a 3 Tr APS and PSs and a CDS-based readout (DSR) circuit that converts a V_SO_ signal of the sensor pixel to the stimulation current I_STIM_.

**Figure 3:**
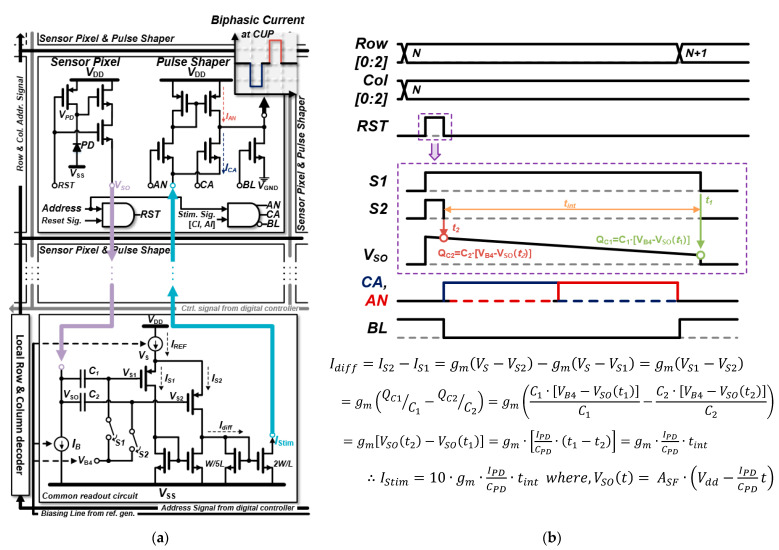
(**a**) Schematic of the pulse shaper and CDS-based readout circuit, which collaborates address signals from the digital controller and bias voltages from the reference generator (gray); (**b**) timing diagram on a particular sequence coupling with a single SPPS pixel. The stimulus current amplitude I_STIM_ is defined in the equation under the timing diagram.

Figure 3a shows the schematics of the SPPS and the DSR circuit with address decoders. An address decoder determines which SPPS is selected to connect with the DSR circuit and is repeated after 25 sequences for cycling SPPSs. A customized photodiode, which has the structure of an N+ and P-sub, is applied due to its high sensitivity [15] and lower fabrication cost than the CMOS image-sensor process [16]. Figure 3b depicts a timing diagram of the SPPS and DSR circuit in a particular sequence of the cycle. When an RST signal turns into logic “1”, the selected SPPS and DSR circuit are coupled, and the PMOS reset transistor of its sensor pixel is turned off. Subsequently, a pre-charged V_PD_ voltage drops according to a photocurrent I_PD_, and the parasitic capacitance of sampling switches S_1_ and S_2_ captures a slope in the dropping V_PD_ in a specific time interval and stores it in sampling capacitors, C_1_ and C_2_, respectively. A branch current of I_B_ after the reset transistor returns to logic “1” is deactivated to diminish unnecessary power dissipation. The stored voltage on the sampling capacitor is used as the differential input of a V-I conversion amplifier in the DSR circuit. Thereafter, the DSR circuit generates the stimulation circuit current I_STIM_ based on the following equation under the timing diagram. Consequently, a biphasic current waveform of the PS, which is referred to as the amplitude of I_STIM_, is generated.

**Figure 4:**
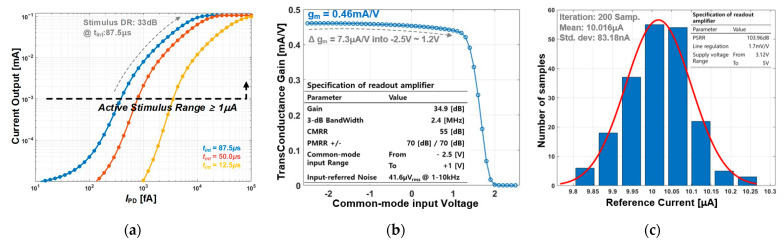
Stimulation results of the proposed retina chip; (**a**) current amplitude versus photocurrent with varying integration time; (**b**) trans-conductance gain of the DSR circuit with a 7.3 μA/V variation in a wide common-mode input range; (**c**) Monte-Carlo simulation result of the reference generator.

Figure 4a shows a current amplitude from the PS corresponding to I_PD_. In this simulation environment, we employed ideal components to model the photodiode, along with its I_PD_ and parasitic capacitor, C_PD_. The maximal current amplitudes of I_CA_ and I_AN_ were given as 0.1 mA, originating from the differential current of I_S1_ and I_S2_ (I_diff_). In this simulation, the time interval between S_1_ and S_2_ and stimulus durations of anodic and cathodic signals, t_ANOD_ and t_CATH_, were set to 87.5 µs and 0.95 ms, respectively. Thus, the frame rate used to complete a cycle of SPPSs was 20 Hz. The dynamic range of the current stimulation within an active stimulus range was estimated as 33 dB and varied with the integration time. Figure 4b shows a trans-conductance gain in a common-mode input range and a specification of the V-I amplifier. The notable specifications of the V-I amplifier were the common–mode–rejection ratio (CMRR) and a power supply rejection ratio (PSRR) to minimize its contribution to the current deviation of I_STIM_. The noise specification of the V-I amplifier was 41.6 µV_rms_, which indicated that the output current noise of the V-I amplifier was 19.136 nA_rms_. Figure 4c shows a Monte-Carlo simulation result of the reference generator that affected the current mismatch of the PS; the standard pixel-to-pixel current deviation was approximately 0.83 μA. 

## 3. Measurement Results

**Figure 5:**
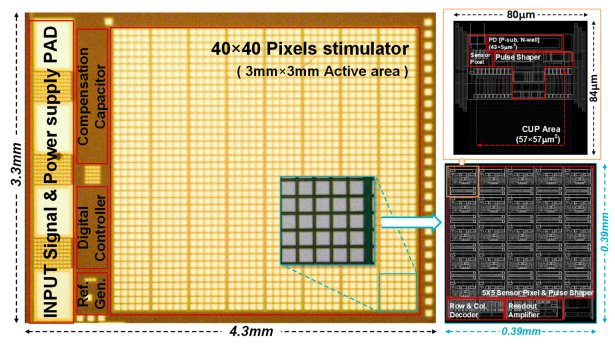
Microscopic image of the proposed retina chip.

Figure 5 shows a micrograph of the 1600-pixel stimulator array adopting the proposed DCR circuit, which was fabricated in a single chip using a standard 0.18 μm 1P6M CMOS process. The retina chip occupies an area of 4.3 mm × 3.3 mm and has an active area generating stimulus patterns of 3 mm × 3 mm.

**Figure 6:**
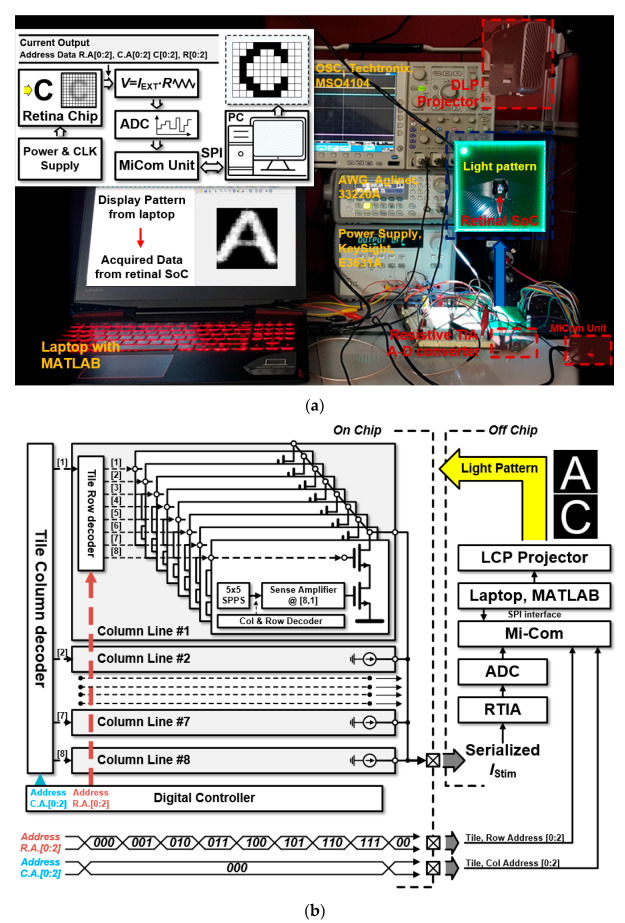
(**a**) Demonstration bench of the proposed retina chip; (**b**) details of the experimental setup with the serialized I_STIM_ and its off-chip circuits as an ADC, and a resistive trans-impedance amplifier (RTIA).

A demonstration bench of the retina chip, as well as its simplified diagram, is depicted in Figure 6a, showing the procedure for measuring a serialized output current from the retinal chip. A tile-selection decoder created a selected tile that connected with a resistive feedback trans-impedance amplifier on an external demonstration bench. The serialized I_STIM_ from the retina chip was digitized by the ADC (ADC104S series, Texas Instrument) that communicated with a micro-computer processor to deliver the digitized data of the I_STIM_ to a laptop. A light pattern from the laptop was implemented using an LCD projector illuminating the center of the packaged retina chip (Figure 6a). 

**Figure 7:**
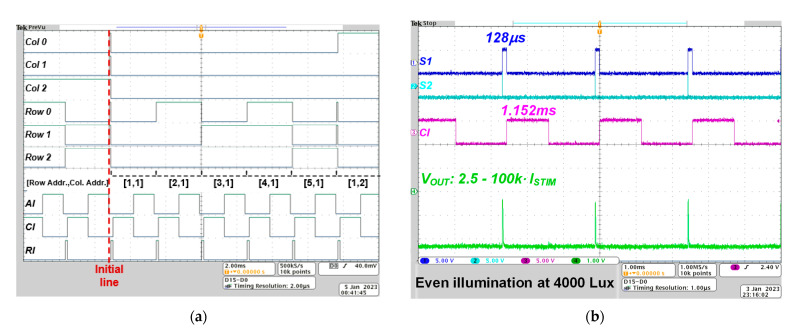
Measurement result of digital signals and current output I_STIM_ of the proposed retina chip; (**a**) oscilloscopic image of the digital signals when changing the address signal; (**b**) oscilloscopic image of the current output when exposing the retina chip to even illumination.

We first performed a benchtop experiment to measure the transient digital signals operating the SPPS and the DSR circuit. Figure 7a presents an oscilloscopic image of digital signals for columns [0:2] and rows [0:2], as well as the durations of anodic current (AI), cathodic current (CI), and integration time (RI). The digital signal underwent sequential changes, transitioning from [1, 1] to [5, 5] as each of the durations ended. When the duration for [5, 5] was completed, the cycle restarted, beginning again with [1, 1]. The durations for AI, CI, and the integration time were controlled by modulating a clock signal that was applied to the digital controller. Figure 7b shows an oscilloscopic image for sampling signals of *S*_1_, *S*_2_, *CI*, and a current amplitude converted using a 100 kΩ resistor. The converted stimulus current in this result was measured using a constant light illuminator (Model: LED-66088, Newport, CA, USA) that eliminated flickering. This result indicated that the temporal resolution of the proposed retina chip can be adjustable in the 1–20 Hz range by managing the stimulus duration and integration time. In addition, under even illumination conditions, the generated current amplitude is almost constant, regardless of the location of the connected SPPS. This means that the proposed retina chip can generate a near-constant stimulus current with low fixed-pattern noise [17]. 

**Figure 8:**
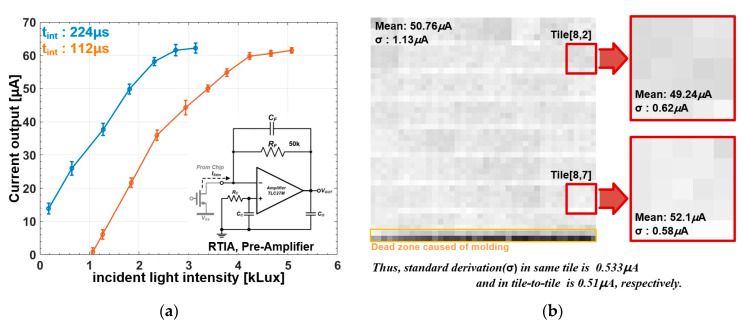
Measurement results of the stimulus current using RTIA pre-amplifier: (**a**) stimulus current varying with incident light intensity; (**b**) stimulus pattern generated through the retina chip.

To evaluate the stimulus current from the proposed retina chip, Figure 8 shows the measurement results of I_STIM_. To compensate for the effect of channel-length modulation, the transistor generating I_STIM_ was combined with a pre-amplifier comprising a commercial operation amplifier (TLC27M, Texas Instrument) and a 50 kΩ feedback transistor. Figure 8a shows the measured current amplitudes relying on the incident light intensity. The experimental results indicated that the stimulus current of the proposed retina chip has a range of from 0 to 63 μA in response to incident light intensities ranging from 1100 to 5100 lux. When the integration time is controlled by enabling the inputs of a 3-bit decoder, the retina chip can operate under variable illumination conditions. The error bars at each step of the markers indicate the pixel-to-pixel variation caused by the input-referred noise and process variation. As a result, the pixel-to-pixel variation is characterized by standard deviations of a few microamperes. Figure 8b shows details of the pixel-to-pixel variation under an even illumination condition when a mean stimulus current of 50.76 μA was generated. The standard deviation in this experimental result was 1.13 μA, indicating that the pixels achieved a smaller standard deviation when they shared the same DSR circuit. The problem of the standard deviation in the same tile is an effect of the input-referred noise from the DSR circuit. However, the overall standard deviation is influenced by the DSR circuit and by a mismatch of the reference current I_REF_ (Figure 3a). Thus, the overall performance of the proposed retina chip is summarized in Table 1. Compared with previous works, this chip can address the high spatial and temporal resolution of artificial vision. Considering the active area, the proposed retina chip contains high-density stimulator pixels. Further, the temporal resolution is adjustable between 1 and 20 Hz, which supports the wide-ranging stimulus period. The overall performance of current deviations characterizes that this proposed retina chip can restore high-quality artificial vision to implanted patients.

## 4. Conclusions

This paper presents a high-temporal and high-spatial-resolution neural stimulator to achieve non-flickering and high-spatial-resolution visual restoration. The neural stimulator array is enabled with a DSR circuit that recognizes light intensity in a short integration time. Additionally, even with a low mismatch in the Monte-Carlo simulation, the retina chip has a small standard deviation of the stimulus current compared with conventional retina chips. A digital controller for producing diverse stimulus period, integration times, and stimulus duration, was integrated into this retina chip. Applying a customized N+/P-sub photodiode in a standard CMOS process facilitates a relatively lower fabrication cost compared with the complex CMOS image sensor process. This 1600-pixel high-resolution retina chip, employing the proposed DSR circuit, was fabricated in a standard 0.18 μm CMOS process and bench-top tested with an LCD projector. As a result, the retina chip achieved a maximum stimulus period of 20 Hz, and a current deviation under 1.2 μA on the standard deviation of the overall stimulus current. For future work, the proposed high-density stimulation chip will be merged with a wireless power transmission system [20] and packaged for clinical trials.

## Figures and Tables

**Table 1:** Specifications of the proposed and state-of-the-art retina chips.

		This Work	TBioCAS’20 [18]	TBioCAS’21 [19]
Target		Sub-Retina	Sub-Retina	Sub-Retina
Fabrication process		0.18 μm	0.18 μm	0.18 μm
Sensor pixel	Image sensor	3Tr-APS	PFM ^(4)^	LSDM ^(4)^
	Power/channel	1.98 μW	56.3 nW	4.49 nW
	Current deviation	≤0.63 µA	≤3 µA	≤6.175 µA
Pulse shaper	Stimulation strategy	Sequential	Simultaneous	Sequential
	Compliance voltage	±2.35 V	±1.6 V	1 V
	Maximum I_STIM_	≤60 µA	≤1 mA	≤95 µA
	Stimulus duration	≥1.152 ms	-	≤15 ms
	Power_MAX_/channel ^(1)^	10.8 µW	1.45 µW	4.28 µW
	Current deviation	≤0.51 µA	-	≤0.475 µA
	Stimulus frequency	1–20 Hz	≤17.06 Hz	4–8 Hz
Area occupation	Size of pixel	84 × 80.3 µm^2^	84 × 86.6 µm^2^	90 × 60 µm^2^
	Active area ^(2)^	3 × 3 mm^2^	3.33 × 3.15 mm^2^	2.85 × 2.55 mm^2^
	Size of chip	4.3 × 3.3 mm^2^	5 × 3.45 mm^2^	2.93 × 2.55 mm^2^
Overall	Number of pixels	1600	1225	288
	Quiescent power ^(3)^	3.45 mW	1.14 mW	-

^(1)^ Power consumption when generating maximum stimulus currents. ^(2)^ Area occupation of stimulation electrodes. ^(3)^ Overall power consumption without generating stimulus current. ^(4)^ PFM and LSDM are pulse-to-frequency modulation and light-to-stimulus duration modulation, respectively.

## Data Availability

No new data were created or analyzed in this study. Data sharing is not applicable to this article.
